# Cognitive impairment influencing factors in the middle-aged and elderly population in China: Evidence from a National Longitudinal Cohort Study

**DOI:** 10.1371/journal.pone.0324130

**Published:** 2025-05-21

**Authors:** Biqi Zu, Ning Wang, Lijun Fan, Jinsong Huang, Yunan Zhang, Meiling Tang, Yulan Wu

**Affiliations:** 1 Department of Psychiatry, Dalian Seventh People’s Hospital, Dalian, China; 2 10th Maternity Ward, Dalian Women and Children’s Medical Group, Dalian, China; 3 Department of Teaching and Research, School of Nursing, Qiqihar Medical College, Qiqihar, China; Lorestan University, IRAN, ISLAMIC REPUBLIC OF

## Abstract

**Objective:**

The study aimed to explore the factors influencing cognitive impairment among middle-aged and elderly individuals in China, utilizing data from the China Health and Retirement Longitudinal Study (CHARLS). By examining the combined effects of socioeconomic factors, health behaviors, chronic diseases, and other multidimensional factors, this research seeks to provide evidence for the development of targeted public health policies and interventions.

**Methods:**

This study utilized data from the 2018 wave of the CHARLS database, including a total of 19,307 participants aged 45 and older. Cognitive impairment was assessed using the Mini-Mental State Examination (MMSE), with thresholds adjusted for education levels. A combination of LASSO (Least Absolute Shrinkage and Selection Operator) regression and logistic regression models was employed to identify significant risk and protective factors for cognitive impairment. RCS (Restricted cubic splines) were used to explore the dose-response relationship between sleep duration and cognitive function.

**Results:**

The analysis identified several significant risk factors for cognitive impairment, including age, urban household registration, self-rated health as fair, chronic disease, impaired instrumental activities of daily living (IADL), alcohol consumption and exercise. Protective factors included sleep duration, being female, divorced or widowed, self-rated health as poor and disability. The study also found that sleep duration followed a U-shaped relationship with cognitive function, with an optimal sleep duration of 5.83 hours per day.

**Conclusion:**

This study highlights the multifactorial nature of cognitive impairment and underscores the importance of early interventions targeting modifiable risk factors such as chronic diseases and lifestyle behaviors. The findings contribute to the growing body of knowledge on cognitive impairment in China’s aging population and provide a basis for evidence-based public health strategies.

## Introduction

With the rapid aging of the global population, cognitive impairment has become an increasingly critical public health issue. According to projections by the United Nations, by 2030, the number of people aged 60 years and older worldwide will reach approximately 1.4 billion, accounting for over 16% of the global population [1]. The incidence of cognitive impairment increases with age, with mild cognitive impairment (MCI) and neurodegenerative disorders such as Alzheimer’s disease becoming increasingly prevalent [2]. According to data from the World Health Organization (WHO), approximately 50 million people globally are living with cognitive impairment, the vast majority of whom have Alzheimer’s disease [3]. The growing size of this population not only affects the quality of life of older adults but also imposes a substantial socioeconomic burden. Consequently, the prevention and management of cognitive impairment has emerged as a pressing global health challenge.

In China, the pace of population aging and the associated risk of cognitive impairment are particularly pronounced. It is projected that by 2035, the number of people aged 60 years and older in China will exceed 400 million, accounting for over 30% of the total population [4]. This demographic shift places China at a heightened risk for cognitive impairment [5]. Recent studies have shown a steady rise in cognitive decline among Chinese older adults, with prevalence increasing from 11% in 2011 to 28% in 2019, particularly in regions with significant urban–rural disparities [6]. Furthermore, due to China’s unique socioeconomic conditions, cultural factors, and family structures, the impact of cognitive impairment on the elderly is more complex and profound. As the affected population continues to grow, cognitive health has become a key issue influencing the trajectory of healthy aging in China [7].

In recent years, a range of interventions targeting cognitive impairment have been implemented in China, including the establishment of elderly health management platforms and cognitive health promotion initiatives. Nevertheless, the prevalence of cognitive impairment continues to rise, which is closely linked to the limitations of current intervention strategies and related research. Specifically, existing studies often suffer from small sample sizes [8,9], limited geographic representation, and a focus on urban or region-specific populations [10–12], thereby failing to reflect the cognitive health status of the nationwide elderly population. Moreover, current interventions primarily emphasize individual-level treatment approaches [13,14], lacking comprehensive analyses of multidimensional influencing factors such as socioeconomic status, health behaviors, and chronic diseases. These methodological shortcomings have contributed to suboptimal intervention outcomes, highlighting the urgent need for a more holistic and integrated research perspective.

In light of the above, this study aims to systematically investigate the multidimensional factors associated with cognitive impairment among middle-aged and older adults in China, using data from the China Health and Retirement Longitudinal Study (CHARLS). In contrast to previous studies, this research adopts a large-scale, nationally representative design, seeking to address existing gaps in the literature and provide robust evidence for the development of targeted public health policies and intervention strategies. By comprehensively analyzing socioeconomic, behavioral, and clinical factors, the study aims to identify effective approaches to improve cognitive health outcomes. Ultimately, it seeks to support policy and practice innovations that enhance the health and quality of life of the aging population in China.

## 1. Data and methods

### 1.1. Data source

The China Health and Retirement Longitudinal Study (CHARLS) is a nationally representative, longitudinal survey aimed at examining the health, economic, and retirement status of Chinese individuals aged 45 years and older, along with their spouses. Conducted from 2011 to 2020, CHARLS stands as the first national survey focused on the middle-aged and elderly population in China, providing a comprehensive, high-quality public micro-database that is valuable for scientific and policy research.

The 2018 wave (Wave 4) of CHARLS, which serves as the data source for this study, included data from 150 county-level units (excluding Tibet) and 450 villages/urban communities across China. The survey utilized a multistage probability sampling method, encompassing 19,744 individuals from 10,257 households. It collected extensive information on various topics, including demographics, health status, economic conditions, family structure, and social support. Additionally, physical measurements, health examinations, and lifestyle factors were also recorded.

This study is a cross-sectional analysis based on the 2018 CHARLS data, with ethical approval obtained from the Biomedical Ethics Committee of Peking University (Approval No: IRB00001052–110155). All participants who consented to take part in the survey signed an informed consent form. The CHARLS survey excludes individuals residing in collective dwellings, such as nursing homes, military bases, and schools, ensuring that the data reflect the experiences of community residents.

By offering a robust and internationally comparable dataset on the health and socioeconomic conditions of the middle-aged and elderly population in China, CHARLS provides valuable insights into the challenges and needs of this demographic group.

### 1.2. Inclusion and exclusion criteria

**Fig 1:**
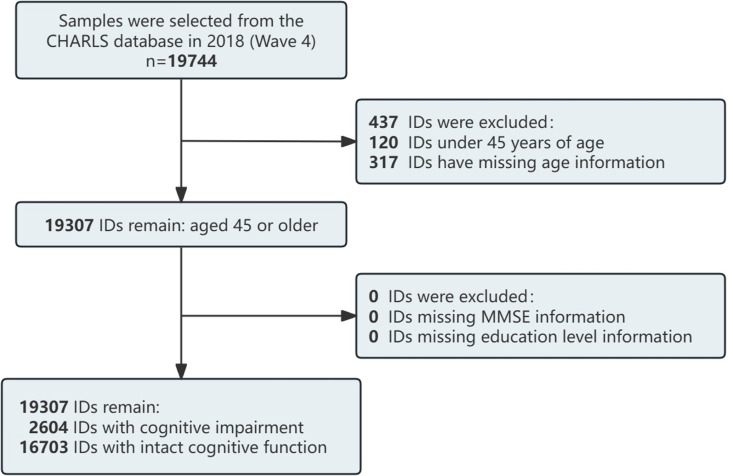
Data filtering flowchart.

Inclusion Criteria: ① Age ≥ 45 years. ② Complete data available for Mini-Mental State Examination (MMSE) score. Exclusion Criteria: Missing information on educational attainment. The data cleaning process is shown in **Fig 1**.

### 1.3. Cognitive function assessment

Cognitive function in this study was assessed using the MMSE [15], a widely recognized tool for evaluating cognitive impairment and intellectual functioning. The MMSE provides a comprehensive and accurate reflection of the degree of cognitive deficits and intellectual levels, serving as a basis for neuropsychological diagnosis and treatment. The MMSE covers multiple cognitive domains, including memory, orientation, comprehension, attention, and reading/constructional abilities. The total score ranges from 0 to 30, with higher scores indicating better cognitive function.

In this study, the criteria for cognitive impairment were adjusted based on participants’ educational levels: a score of ≤17 for illiterate individuals, ≤ 20 for those with primary education, and ≤24 for those with secondary education or higher was classified as cognitive impairment, while scores above these thresholds were considered indicative of normal cognitive function [16].

### 1.4. Variable selection and definition

Based on empirical evidence and database information, a total of 14 variables were selected for inclusion in the LASSO regression for variable selection: Gender, Age, Sleep Duration, Marital Status, Residential Address, Self-Rated Health, Chronic Disease, ADL (Activities of Daily Living), IADL (Instrumental Activities of Daily Living), Alcohol Consumption, Smoking Status, Exercise Status, Disability Status, and Social Activities.

The Self-Rated Health question is “How do you perceive your health status?” with possible responses being “Excellent”, “Very Good”, “Good”, “Fair”, and “Poor”. Responses of “Excellent”, “Very Good”, and “Good” were combined and categorized as “Good”, while the responses of “Fair” and “Poor” remained unchanged.

Chronic Disease were based on the answer to the question in the CHARLS questionnaire: “Has a doctor ever diagnosed you with any of the following chronic conditions?” The chronic conditions include: Hypertension, Dyslipidemia, Diabetes or Elevated Blood Sugar (including abnormal glucose tolerance or elevated fasting blood sugar), Cancer or Malignant Tumors, Chronic Pulmonary Diseases (such as chronic bronchitis or emphysema), Pulmonary Heart Disease (excluding tumors or cancer), Liver Disease (excluding fatty liver, tumors, or cancer), Heart Disease (such as myocardial infarction, coronary heart disease, angina, congestive heart failure, and other heart diseases), Stroke, Kidney Disease (excluding tumors or cancer), Gastrointestinal or Digestive System Diseases (excluding tumors or cancer), Emotional and Mental Disorders, Memory-Related Diseases (such as Alzheimer’s Disease, Brain Atrophy), Parkinson’s Disease, Arthritis or Rheumatism, and Asthma (non-pulmonary disease), totaling 14 conditions.

The definition of ADL (Activities of Daily Living) includes the following six items: Difficulty with Dressing, Difficulty with Bathing or Showering, Difficulty with Eating, Difficulty with Getting into or out of Bed, Difficulty with Using the Toilet, and Difficulty with Controlling Urination and Defecation. If no difficulties are reported in any of these areas, the individual is categorized as “Normal.” If any difficulties are reported in one or more of these areas, the individual is categorized as “Abnormal.”

The definition of IADL (Instrumental Activities of Daily Living) includes the following six items: Difficulty with Household Chores, Difficulty with Preparing Hot Meals, Difficulty with Shopping for Groceries, Difficulty with Taking Medications, Difficulty with Managing Money, and Difficulty with Making Phone Calls. If no difficulties are reported in any of these areas, the individual is categorized as “Normal.” If any difficulties are reported in one or more of these areas, the individual is categorized as “Abnormal.”

The definition of Exercise Status in this study categorizes “Exercises Regularly” and “Occasionally Exercises” as “YES,” while “Does Not Exercise” is categorized as “NO.”

The definition of disability in this study is based on the CHARLS database, which includes the following categories: Physical Disabilities, Brain Damage/Mental Retardation, Vision Problem, Hearing Problem, and Speech Impediment. In this study, if the sample includes a response of “yes” to any of these categories, it is categorized as “Disability.” Otherwise, it is defined as “No Disability.”

The investigation of Social Activities is based on a subjective questionnaire that includes 11 multiple-choice questions and one open-ended question. It asks participants about the activities they have engaged in during the past month, including: Interacted With Friends, Played Ma-jong, Played Chess, Played Cards, or Went to, Provided Help to Family, Friends, or Neighbors who Do Not Live with You, Went to a Sport, Social, or Other Kind of Club, Took Part in a Community-Related Organization, Done Voluntary or Charity Work, Cared for a Sick or Disabled Adult who Does Not Live With You, Attended an Educational or Training Course, Stock Investment, Used the Internet, Other, or None of These. Social Activities were defined as “None” if a participant did not engage in any of the activities mentioned in the questionnaire. No changes were made to the other variables during data extraction and processing.

## 2. Data processing and statistical methods

### 2.1. Missing variables and data handling

Some of the independent variables in the included samples had missing data, as reported below: Residential Address had 287 (1.48%) missing values; Sleep Duration had 30 (0.15%) missing values; ADL (Activities of Daily Living) had 5999 (31.07%) missing values; IADL (Instrumental Activities of Daily Living) had 38 (0.19%) missing values; Smoking Status had 33 (0.17%) missing values; Exercise Status had 30 (0.15%) missing values; Disability Status had 26 (0.13%) missing values.

Multiple imputation methods were used to handle the missing data for these indicators and other variables. Five imputed datasets were constructed based on the original dataset. The Rubin formula was applied to obtain model estimates and compare the corrected standard errors of the imputations [17].

### 2.2. Statistical analysis

Categorical data were presented as frequencies and proportions, and differences between groups were assessed using the chi-square test.

Due to the inclusion of numerous variables and the high dimensionality of the data, this study employed a combination of LASSO regression and logistic regression for multivariate analysis of cognitive status.

LASSO regression, a penalized regression technique, was used to select the most relevant variables by shrinking the coefficients of less important variables to zero, thereby retaining only those strongly associated with cognitive status. A seed number (123) was set to ensure reproducibility, and the “glmnet” package in R was used for the analysis. The optimal lambda (λ) value was determined using 10-fold cross-validation. Variables with non-zero coefficients at λ.min were selected and incorporated into a multivariate logistic regression model. RCS were plotted using the ‘rms’ package to adjust for confounding factors such as Age and Gender. Logistic regression models with RCS were constructed to explore the relationship between sleep duration and cognitive function and to calculate the optimal sleep duration.

All statistical analyses were conducted using R software (version 4.3.3), and results were considered statistically significant with *P*-values less than 0.05.

## 3. Results

### 3.1. General population characteristics

A total of 19,307 participants were included in this study, with 9,189 males (47.5%) and 10,118 females (52.5%). The overall prevalence of cognitive impairment was 13.48% (2,604/19,307). The proportion of males with Cognitive Impairment was higher (15.9%). The average age of 19,307 participants was 63.88 ± 10.17 years. Detailed demographic characteristics of the study participants are shown in **Table 1**.

**Table 1:** Study Subjects General Population Characteristics (n, %).

Variable	Total (n = 19,307)	Cognitive Intact (n = 16,703)	Cognitive Impairment (n = 2,604)	χ² Value	*P*-value
**Gender**				90.617	1.74*10^−21^
Male	9,189 (47.5)	7,724 (84.1)	1,465 (15.9)		
Female	10,118 (52.4)	8,979 (88.7)	1,139 (11.3)		
**Age**				1913.545	2.22*10^−16^
45-59 years	7,567 (39.2)	7,560 (99.9)	7 (0.1)		
≥60 years	11,740 (60.8)	9,143 (77.9)	2,597 (22.1)		
**Marital Status**				0.710	0.70
Married	16,549 (85.7)	14,317 (86.5)	2,232 (13.5)		
Unmarried	111 (0.6)	99 (89.1)	12 (10.9)		
Divorced or Widowed	2,647 (13.7)	2,287 (86.3)	360 (13.7)		
**Educational Level**				511.422	8.83*10^−112^
≤ Primary School	8,263 (42.7)	6,920 (83.8)	1,343 (16.2)		
Junior High School	4,321 (22.4)	3,470 (80.4)	851 (19.6)		
≥High School	6,723 (34.9)	6,313 (94.0)	410 (6.0)		
**Residential Address**				49.479	1.80*10^−11^
Rural	14,316 (74.2)	12,517 (87.4)	1,799 (12.6)		
Urban	3,519 (18.2)	2,918 (82.9)	601 (17.1)		
Other	1,472 (7.6)	1,268 (86.1)	204 (13.9)		

### 3.2. LASSO regression selection results

**Fig 2:**
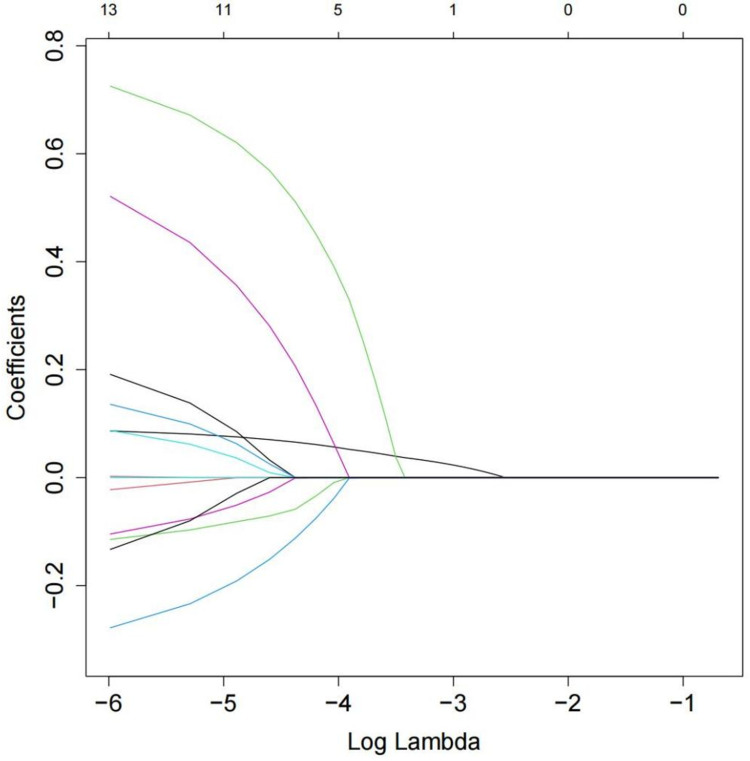
The best λ value was selected by 10-fold cross-validation-coefficient shrinkage plot.

**Fig 3:**
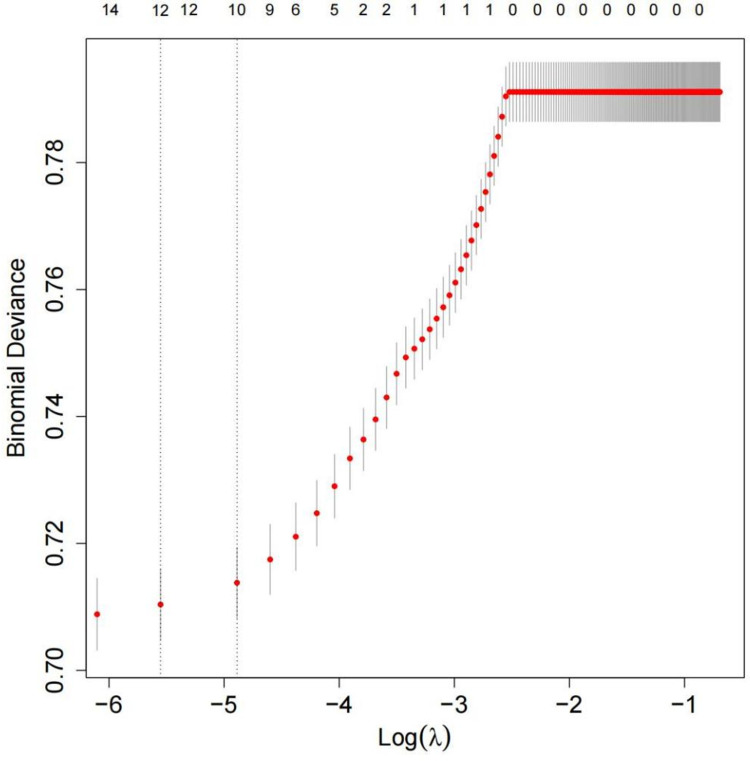
Cross-validation plot. Note: Two vertical lines denote lambda. min and lambda.1se (Fig 3), with lambda. min representing the λ value at minimum model error and lambda.1se representing the λ value within one standard error of the minimum model error.

Under 10-fold cross-validation of the LASSO regression, as the penalty coefficient increased, the variables gradually shrank and reduced (**Fig 2**). In this study, variables with non-zero coefficients at the minimum λ value (**Fig 3**) were selected for logistic regression analysis. At this point, λ.min = 2.82 × 10^−6^, and the variables with non-zero coefficients included 12 factors: Gender, Age, Sleep Duration, Marital Status, Residential Address, Self-Rated Health, Chronic Disease, ADL, IADL, Alcohol Consumption, Exercise Status, and Disability Status.

### 3.3. Multivariate logistic regression analysis results

Cognitive impairment in middle-aged and older adults (0 = cognitively intact, 1 = cognitively impaired) was used as the dependent variable, and the 12 variables selected by LASSO regression were used as independent variables in a multivariate logistic regression analysis. The forward and backward stepwise method was used to train the model. The results are shown in **Table 2**. Age, Residential Address (Urban), Self-Rated Health (Fair), Chronic Disease, impaired IADL, Alcohol Consumption, and Exercise were identified as risk factors for cognitive impairment. Sleep Duration, Female, Divorced or Widowed, Self-Rated Health (Poor), and Disability Status (Yes) were identified as protective factors for cognitive impairment. Unmarried status and ADL status were not associated with cognitive function.

**Table 2:** The variables selected by lasso regression were included in the multivariate logistic regression analysis.

Variables	B	SE	Wald χ^2^	*OR* (*95% CI*)	*P*-value
**Age**	0.093	0.003	1251.876	1.098 (1.092–1.103)	<0.001
**Sleep Duration**	−0.037	0.011	10.921	0.964 (0.943–0.985)	0.001
**Gender (Female)**	−0.116	0.051	5.229	0.891 (0.806–0.984)	0.022
**Marital Status**					
*Married*			85.241	*Ref*	<0.001
*Unmarried*	−0.185	0.322	0.33	0.831 (0.442–1.563)	0.566
*Divorced or Widowed*	−0.649	0.07	85.127	0.523 (0.455–0.600)	<0.001
**Residential Address**					
*Rural*			12.086	*Ref*	0.002
*Urban*	0.183	0.056	10.818	1.201 (1.077–1.339)	0.001
*Other*	0.138	0.084	2.707	1.148 (0.974–1.352)	0.100
**Self-Rated Health**					
*Good*			49.414	*Ref*	<0.001
*Fair*	0.208	0.067	9.579	1.231 (1.079–1.404)	0.002
*Poor*	−0.159	0.076	4.458	0.853 (0.735–0.989)	0.035
**Chronic Disease (YES)**	0.244	0.045	29.148	1.277 (1.168–1.395)	<0.001
**ADL (Impairment)**	0.045	0.062	0.525	1.046 (0.926–1.181)	0.469
**IADL (Impairment)**	0.739	0.061	145.021	2.094 (1.857–2.362)	<0.001
**Alcohol Consumption (YES)**	0.171	0.054	10.046	1.187 (1.068–1.320)	0.002
**Exercise Status (YES)**	0.598	0.086	48.622	1.819 (1.537–2.152)	<0.001
**Disability Status (YES)**	−0.165	0.05	10.835	0.848 (0.769–0.936)	0.001

NOTE: *SE*: Standard Error; *OR*: Odds Ratio; *CI*: Confidence Interval.

### 3.4. The results of the RCS analysis

**Fig 4:**
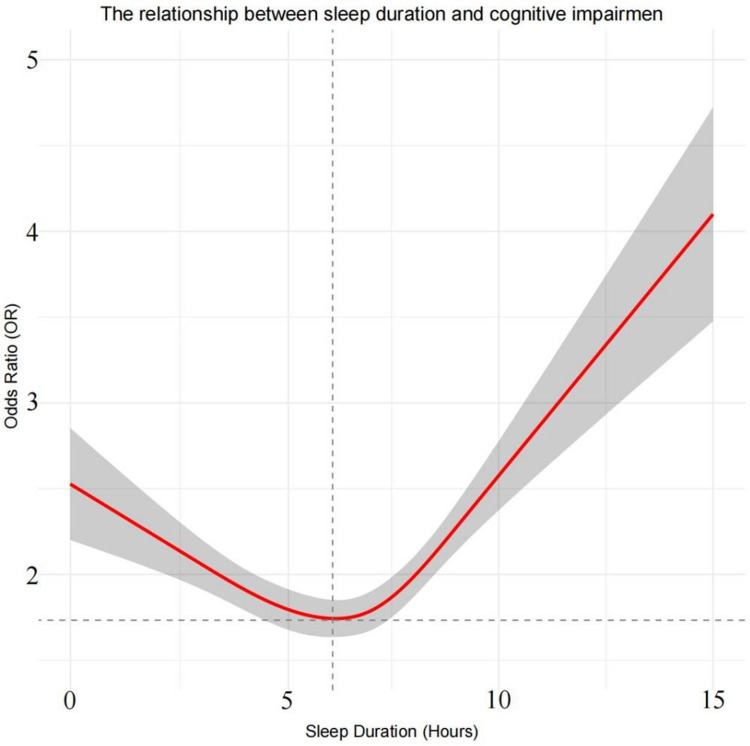
The relationship between sleep duration and cognitive impairment after adjusting for confounding factors.

Based on previous experience, we believe that the linear relationship between sleep duration and cognitive function (*OR* = 0.964, *95% CI*: 0.943–0.985) may not hold. In other words, the likelihood that longer sleep duration results in better cognitive function may not necessarily be true. Therefore, we used RCS to model the relationship between sleep duration and cognitive function, setting the number of knots to 4. The results, shown in **Fig 4**, indicate that the impact on cognitive function is lowest when the sleep duration is 5.83 hours per day. *P*_overall_<0.05, *P*_nonlinear_ = 0.044.

## 4. Discussion

The results of the study indicate that age is a significant risk factor for cognitive impairment, with an *OR* of 1.098 (*95% CI*: 1.092–1.103). Specifically, for each additional year of age, the risk of cognitive impairment increases by approximately 9.8%. Previous studies [18] have supported a close relationship between age and cognitive decline. As age increases, physiological changes in the body and brain lead to a decline in various cognitive functions. Age-related neurodegenerative diseases, such as Alzheimer’s disease (AD) and other forms of cognitive impairment, tend to exacerbate with advancing age. The mechanisms behind these neurodegenerative diseases are closely related to changes in brain structure and function. Research has shown [19] that as age increases, neural cells in the brain gradually lose function, and neural transmission processes slow down, which can lead to a decline in cognitive functions such as attention, memory, and language skills. Furthermore, aging can result in other physiological and biochemical changes, such as reduced blood flow, insufficient oxygen supply, and a decline in neurotransmitter function, all of which can further affect cognitive function in the brain [20]. In addition to biological mechanisms, aging is also accompanied by changes in socio-psychological factors that may indirectly affect cognitive health. For example, older adults may face increased loneliness and social isolation, both of which have been identified as significant risk factors for cognitive decline [21]. Additionally, the widespread use of medications and changes in lifestyle (such as reduced physical activity and poor nutrition) in the elderly population may further accelerate the decline in cognitive function [22].

The study shows that sleep duration is a significant factor influencing cognitive function, with an *OR* of 0.964 (*95% CI*: 0.943–0.985), meaning that for each additional hour of sleep, the risk of cognitive impairment decreases by approximately 3.6%. This result highlights the significant role of sleep in protecting cognitive function, particularly in individuals with insufficient sleep, where increasing sleep duration helps reduce the risk of cognitive decline. However, the restricted cubic spline analysis indicates that when daily sleep duration increases from 0 to 5.83 hours, its effect size on cognitive risk has decreased. Once sleep duration exceeds 5.83 hours, the risk of cognitive impairment increases as sleep duration increases. This U-shaped relationship suggests that the association between sleep duration and cognitive function follows a more complex dynamic pattern. Moderate sleep has significant benefits for brain health, whereas excessive sleep may indicate other underlying health issues that could negatively affect cognitive function [23,24]. Sleep deprivation prevents the brain from receiving adequate rest and repair, and prolonged sleep deprivation can affect brain structure and function, increasing the risk of cognitive decline. Sufficient sleep is essential for brain health, especially during the repair and memory consolidation processes that occur at night. Studies have shown that the deep sleep phase is critical for the brain to clear waste and repair damage, particularly in reinforcing and repairing neural cells and connections [25]. Additionally, sleep helps enhance learning and memory processes; especially after learning new information, sleep aids in converting short-term memories into long-term ones, thereby improving cognitive function. On the other hand, excessive sleep has also been linked to cognitive impairment. Chen W’s study [24] suggests that too much sleep may lead to daytime sleepiness, difficulty concentrating, and memory decline, thereby affecting an individual’s cognitive function. Excessive sleep could be indicative of underlying health issues, particularly related to mental health disorders (such as depression and anxiety) or neurodegenerative diseases (such as Alzheimer’s disease). In such cases, although sleep duration is longer, its quality may be poor, or it may be intertwined with other health problems, potentially negatively impacting cognitive function.

According to the study results, females have a lower risk of cognitive impairment compared to males, with an *OR* of 0.891 (*95% CI*: 0.806–0.984). This finding is consistent with related literature and theories. Many studies suggest that gender may influence the incidence of cognitive decline, with females generally having a lower risk of cognitive decline than males [26,27]. This may be attributed to factors such as females’ long-term life experiences, social support, and relatively higher education levels and health behaviors during certain periods. Additionally, some studies [28,29] have pointed out that physiological characteristics of females, such as longer life expectancy and hormonal changes, may also impact cognitive health. However, despite the gender differences, the risk of cognitive impairment is influenced by the interaction of numerous factors, including social, cultural, and psychological factors. Females in many cultures may enjoy more social support and social engagement, which have been shown to contribute to the maintenance of cognitive health.

Compared to married individuals, the risk of cognitive impairment in divorced or widowed individuals is significantly reduced, with an *OR* of 0.523 (*95% CI*: 0.455–0.600). Divorce or widowhood may prompt individuals to reassess their lifestyle, leading them to adopt more proactive health behaviors, such as engaging in more social activities and exercise, which contribute to the maintenance of cognitive health [30]. Moreover, divorced or widowed individuals may rely more on external social support and community resources, which could potentially reduce the risk of cognitive decline. Therefore, although divorce or widowhood is generally considered a negative factor that may increase the risk of cognitive impairment, certain factors in these groups may help mitigate this risk.

Compared to individuals with a rural household registration, those with an urban household registration (*OR* = 1.201, *95% CI*: 1.077–1.339) was found to be at higher risk for cognitive impairment. This conclusion is supported by related studies. For example, XIANG Y et al. found that urban residents are more likely to experience a higher risk of cognitive decline and dementia compared to rural residents [31]. This may be attributed to factors such as increased life stress, lower social support, environmental pollution, and a higher prevalence of chronic diseases in urban environments. The intensification of urbanization is often associated with lifestyle changes, such as poor dietary habits, lack of exercise, and higher psychological stress, all of which could accelerate cognitive decline. Moreover, high population density in urban areas may lead to greater social isolation, further impacting cognitive health [32]. This could explain the association between urban household registration and cognitive impairment.

Compared to individuals with self-rated health as “Good,” those who rated their health as “Fair” had a higher risk of cognitive impairment, with an *OR* of 1.231 (*95% CI*: 1.079–1.404). This result is consistent with studies by Bendayan and colleagues, which also found that poorer self-rated health is associated with an increased risk of cognitive decline, especially in the elderly population [33]. Self-rated health is an important subjective measure of an individual’s overall perception of their physical and mental health. Individuals who rate their health as poor tend to experience more physical and mental health problems, including chronic diseases, poor quality of life, and social isolation, all of which may contribute to cognitive decline. However, a self-rated health of “Poor” does not necessarily predict cognitive decline, as it may also be influenced by an individual’s sensitivity to their health condition and their ability to improve health behaviors (e.g., actively seeking medical help or making lifestyle changes). Therefore, these individuals may, in some cases, demonstrate better cognitive function. Ramsingh also noted that poor self-rated health may prompt individuals to pay more attention to health management, potentially reducing the risk of cognitive decline [34].

The results show that chronic disease is a risk factor for cognitive function, with an *OR* of 1.277 (*95% CI*: 1.168–1.395). Individuals with chronic diseases are at higher risk for cognitive impairment. This conclusion is supported by numerous studies. ANTAL B et al. reported that chronic diseases may affect brain health through various mechanisms, thus accelerating the onset of cognitive decline [35]. For instance, high blood glucose levels caused by diabetes can lead to vascular damage and microvascular changes in the brain. These vascular issues may interfere with brain blood supply, thereby affecting brain function and the maintenance of cognitive abilities. Chronic diseases are also typically associated with systemic inflammation, and this chronic low-grade inflammation is considered harmful to the brain, potentially leading to cognitive decline by damaging neurons and synaptic connections. Moreover, LEE Y’s research found that individuals with chronic diseases, especially the elderly with multiple comorbidities, face a higher risk of cognitive decline [36]. The poor cardiovascular health of chronic disease patients leads to insufficient oxygen and nutrient supply to the brain, which is also a significant factor in cognitive impairment. Additionally, lifestyle and health behaviors of individuals with chronic diseases often exacerbate the deterioration of cognitive function. Studies have shown [37] that psychological factors and lack of social support directly affect the occurrence of chronic diseases, indirectly influencing cognitive function.

Impairment of IADL is a risk factor for cognitive function, with an *OR* of 2.094 (*95% CI*: 1.857–2.362). IADL evaluates an individual’s ability to independently perform complex tasks in daily life, such as managing finances, cooking, shopping, and using transportation, all of which typically require higher cognitive and executive function. Therefore, IADL impairment can be a sensitive indicator of cognitive decline, especially in the elderly population. The relationship between IADL impairment and cognitive decline has been confirmed by several studies. For example, POPE C et al. found that IADL impairment often occurs in the early stages of cognitive decline and can serve as an important predictor of cognitive deterioration [38]. As cognitive function declines, individuals typically experience difficulties in handling complex daily tasks, such as managing finances, household chores, and maintaining social activities. These tasks depend on an individual’s working memory, attention, planning, and decision-making abilities. In addition, IADL, as the factor with the highest effect size on cognitive function, may be related to the fact that the personal abilities required to complete IADL tasks are highly similar to the items assessed by the MMSE scale. For example, managing finances is related to memory and calculation ability, while shopping is related to orientation. Therefore, when IADL is impaired, it often indicates problems with the brain’s executive functions, which in turn suggests a decline in cognitive abilities.

Alcohol consumption is a risk factor for cognitive function, with an *OR* of 1.187 (*95% CI*: 1.068–1.320). Excessive alcohol consumption has been shown to be closely related to an increased risk of cognitive decline and dementia. For instance, WOODS A’s research found that individuals who engage in long-term heavy drinking are more likely to develop cognitive impairment, including Alzheimer’s disease, compared to non-drinkers or moderate drinkers [39]. Even moderate alcohol consumption may be associated with cognitive decline, suggesting that the impact of alcohol on cognitive health may be cumulative. BRENNAN S’s research pointed out that although small amounts of alcohol may have some cardiovascular protective effects, its impact on cognitive function is not entirely harmless, especially in the context of long-term alcohol use [40]. The damage to the brain caused by alcohol primarily manifests as neurotoxicity. Alcohol can affect the brain’s neurotransmitters, damage neuron structures, and reduce neural plasticity, leading to cognitive decline. Alcohol’s metabolic products (such as acetaldehyde) can damage the hippocampal region of the brain, which is crucial for memory and learning abilities, thus leading to symptoms such as memory loss, difficulty concentrating, and executive dysfunction in long-term drinkers.

Exercise is considered a risk factor for cognitive function, with an *OR* of 1.819 (*95% CI*: 1.537–2.152). This result seems contradictory because exercise is generally viewed as a protective factor for cognitive health. However, exercise may reflect that individuals with cognitive impairment attempt to improve their health through physical activity rather than directly causing cognitive decline. JIN Q suggested that exercise in the early stages of cognitive decline might only be a sign of deteriorating health [41]. Additionally, differences in the type and intensity of exercise may affect the results. Some high-intensity exercise could increase physical stress, affecting cognitive health. TAYLOR J’s study indicated that excessive exercise could lead to physical fatigue and hormonal fluctuations, which in turn may affect brain function [42]. Exercise may also indirectly influence cognitive function by affecting the stress response mechanisms (such as cortisol levels), particularly in individuals with chronic health issues or in the elderly population. An individual’s motivation and psychological factors related to exercise can also influence the outcome. MANDOLESI L emphasized that exercise habits, frequency, and psychological motivation are key factors. If individuals feel difficulty or excessive stress during exercise, it may negatively impact their cognitive health [43].

Physical disability is considered a protective factor for cognitive function, with an *OR* of 0.848 *(95% CI*: 0.769–0.936), a result that contradicts the usual negative correlation between disability and cognitive decline. Individuals with disabilities may compensate for functional deficits through cognitive and behavioral strategies. For example, individuals who have lost sensory functions may enhance the use of other senses, which could improve cognitive abilities in other domains. Moreover, disability may prompt individuals to rely more on memory, planning, and decision-making functions, thereby delaying cognitive decline. Disabled individuals typically receive more medical and social support, which benefits cognitive health. MATTKE S’s research suggested that frequent medical examinations and cognitive assessments could help detect cognitive issues early and take preventive measures [44]. Additionally, individuals with disabilities may place greater emphasis on health management and self-care, reducing the risk of cognitive decline. AI F’s research found that social interactions and daily support networks, especially assistance from family and friends, play a vital role in enhancing cognitive health. Psychological adaptation is also an important factor. A positive psychological state and emotional regulation protect cognitive function, helping individuals maintain better social engagement and reduce the risk of cognitive decline [45].

### Limitations

Despite the comprehensive nature of this study, several limitations should be acknowledged. First, the data used in this research is cross-sectional in nature, which limits our ability to establish causality between the identified risk and protective factors and cognitive impairment. Longitudinal studies would be needed to better understand the temporal relationships and causal mechanisms involved in cognitive decline.

Second, while the CHARLS database provides valuable nationwide data, certain subgroups within the elderly population, such as those from remote rural areas or those with severe cognitive impairment, may be underrepresented. This could potentially limit the generalizability of the findings to the entire elderly population of China, particularly those with more severe cognitive impairment or those who are less likely to participate in surveys.

Third, despite the rigorous methods used to handle missing data, such as multiple imputation, there is still a possibility that missing data may introduce bias. Although we attempted to minimize this impact, further validation of the results with datasets from other regions or international cohorts would provide additional insight into the robustness of the findings.

Lastly, while this study identified several factors associated with cognitive impairment, the complex interaction between biological, psychological, and social factors may not have been fully captured. Future research could benefit from incorporating more detailed psychological and neurobiological assessments to deepen the understanding of these relationships.

## Conclusion

In conclusion, this study provides valuable insights into the factors influencing cognitive impairment among middle-aged and elderly individuals in China. The findings highlight the significant roles of age, chronic disease, sleep duration, gender, marital status, residential address, and self-rated health in determining the risk of cognitive impairment. These results underscore the multifactorial nature of cognitive decline and emphasize the importance of early intervention and targeted health policies aimed at mitigating these risks.

This study contributes to the growing body of research on cognitive health in the elderly population and provides a solid foundation for future studies on effective interventions. As China continues to face an aging population, understanding the diverse factors influencing cognitive health is crucial for the development of evidence-based policies and strategies to support the aging population and improve their quality of life.
